# Problem-based learning for in-service training on breastfeeding in Friuli Venezia Giulia, Italy

**DOI:** 10.1186/s13006-021-00439-4

**Published:** 2021-11-27

**Authors:** Emanuelle Pessa Valente, Adriano Cattaneo, Maria Vittoria Sola, Laura Travan, Sofia Quintero Romero, Mariarosa Milinco, Cinzia Decorti, Roberta Giornelli, Cinzia Braida, Patrizia Dalmin, Manuela Giangreco, Luca Ronfani, for the FVG Working Group for Instructional Design on Breastfeeding Training, Julia Bomben, Maria Chiara Calligaris, Giada Casetta, Enrica Causin, Franca Crevatin, Francesca Demitri, Sara Marocco, Graziella Nassimbeni, Isa Piasentin, Maria Vittoria Sola, Marta Pigat, Carla Pittini, Laura Travan

**Affiliations:** 1 WHO Collaborating Center for Maternal and Child Health, Institute for Maternal and Child Health - IRCCS “Burlo Garofolo”, Trieste, Italy; 2 Epidemiologist, Trieste, Italy; 3 Azienda Sanitaria Universitaria Giuliano Isontina (ASU GI), Trieste, Italy; 4 Neonatal Intensive Care Unit, Institute for Maternal and Child Health, Institute for Maternal and Child Health - IRCCS “Burlo Garofolo”, Trieste, Italy; 5 Lactation Consultant, Trieste, Italy; 6 Clinical Epidemiology and Public Health Research Unit, Institute for Maternal and Child Health - IRCCS “Burlo Garofolo”, Trieste, Italy; 7 Centre for Training Activities, Institute for Maternal and Child Health - IRCCS “Burlo Garofolo”, Trieste, Italy; 8 Health Promotion and Prevention, Regional Health Directorate, Trieste, Italy

**Keywords:** Problem-based learning, Problem based curriculum, Continuing medical education, In-service training, Breastfeeding, Italy

## Abstract

**Background:**

Problem-Based Learning (PBL) is extensively used in pre- and post-graduate teaching programmes. However, it has been seldom used for in-service training and continuing medical education. We aimed to develop a PBL curriculum for a short in-service training on breastfeeding for maternal and child health professionals, and to assess the effect of these courses on their knowledge and skills. Also, the project aimed at increasing exclusive breastfeeding rates and duration in an Italian region.

**Methods:**

After initial training on PBL and an assessment of the learning needs of about 400 health professionals, a small working group developed learning objectives, designed a curriculum, produced manuals, and shaped assessment tools for a new PBL course on breastfeeding. The field test of the new course allowed selection of the tutors for the scaling up of the training to the whole region. During this extension phase, participants were asked to complete an evaluation questionnaire. In addition, the health professionals who attended the PBL courses in 2019 were asked to complete an online survey to assess knowledge, attitudes and practices (KAP) just before, soon after the course, and 4–6 months later.

**Results:**

The new 29 − hour PBL course, to be delivered in four days over four consecutive weeks, gives priority to tutorial groups and practical activities (71% of the total time). Supervised clinical practices absorb 16% of time. Ethics, communication and woman-centred clinical management content run throughout the four days and all activities. The three manuals, for tutors, participants and practical activities, facilitate the tasks and performance of tutors and participants. After the field test, 32 regional tutors ran courses for 562 health professionals. The analysis of the evaluation showed a high level of satisfaction for perceived effectiveness, relevance to practice, and educational quality. The KAP questionnaires indicated a general improvement after the course and retention after 4–6 months.

**Conclusions:**

Despite some predictable shortcomings, this new PBL approach for short in-service training courses on breastfeeding showed encouraging results as far as participants’ satisfaction and KAP are concerned. The possible effects on rates and duration of exclusive breastfeeding need further research.

**Supplementary Information:**

The online version contains supplementary material available at 10.1186/s13006-021-00439-4.

## Background

In Italy, the 2008 national policy on the protection, promotion and support of breastfeeding recommends that infants be exclusively breastfed to six months and that breastfeeding be continued, with adequate complementary foods, to one year and beyond or until wished by mothers and infants [1]. Breastfeeding practices, however, fall short of these recommendations. Initiation of breastfeeding increased from 81 to 86% between 2000 and 2013, but the mean duration was limited to 8.3 months of age and only 43% of infants less than six months of age (49% at 0–1, 44% at 2–3, and 39% at 4–5 months of age) were exclusively breastfed [2]. The Italian Baby Friendly Hospital Initiative (BFHI), launched in 2001, does not seem to have taken off. To date (September 2021), only 30 out of about 500 maternity hospitals achieved the BFHI accreditation [3], without having significant effects on national breastfeeding statistics. Health professionals working in hospitals and communities, BFHI accredited or not, usually attend in-service training based on WHO and UNICEF 40 − hour and 18 − hour courses [4, 5].

The 2014–18 National and Regional plan for Health Prevention called for enhanced actions in order to boost exclusive breastfeeding (EBF) rates and duration. In Friuli Venezia Giulia (FVG), a region in the northeast of Italy, the Regional Health Authority decided to promote in-service training on breastfeeding and the FVG group in charge of in-service training opted for an innovative approach: Problem-Based Learning (PBL). The decision was based on an initial review of the benefits of active learning [6–8] and educational methodologies that promotes contextual learning, collaboration and critical thinking [9–14]. The PBL process is facilitated by trained tutors, and it involves interaction and collaboration with colleagues, definition of the problems, individual searching for information, sharing of ideas on possible causes of the problems, and identification and testing of solutions taking into account the clinical and social contexts [10–13].

PBL is extensively used in pre- and post-graduate medical and nursing teaching programmes worldwide. However, it has been seldom used for in-service training and Continuing Medical Education (CME) [15, 16]. In trials on management of mental health problems for occupational health physicians [14], of asthma for primary care physicians [17], and on nursing ethics education [18], for example, PBL was as effective as conventional teaching methods in knowledge uptake and retention but appeared to be more effective in improving performance and stimulating critical thinking [13].

Based on the above principles and evidence, on an integrative literature review that endorsed the use of self-directed learning, interactive techniques and multi-professional learning groups for CME [7, 19–21], and on a perspective article that encouraged saying goodbye to lectures to enhance problem-solving skills [15], the FVG Health Authority decided to create a Regional Working Group for Instructional Design on Breastfeeding Training and to finance and run, in collaboration with the CME unit of the Institute for Maternal and Child Health IRCCS Burlo Garofolo, a regional project for the application of the PBL approach for in-service inter-professional training on the protection, promotion and support of breastfeeding. The aims of the project were to develop a PBL curriculum, and related learning materials, for a short in-service training on breastfeeding, to train with PBL in-service courses all maternal and child health professionals in FVG, and to assess the effect of these PBL courses on their knowledge and skills. As a longer-term objective, the project aimed to increase EBF rates and duration.

## Methods

### Setting

FVG is a region with little more than 1.2 million people (2019), almost 8000 live births (2019), nine maternity hospitals and three Local Health Authorities (LHA) in charge of community health services. Since 1998, FVG has had a reporting system on the prevalence of breastfeeding at discharge from maternity hospitals, where almost 100% of births are assisted, and at four to five months of age, the time when infants attend for their second vaccination [22]. EBF rates were stable since 2016 (around 71% at discharge and around 30% at second vaccination) [23, 24] and the region has two hospitals and one LHA recognized as Baby Friendly by UNICEF Italy.

### Design

This was a longitudinal study including several development, implementation and assessment phases.

### Theoretical framework

In PBL programmes, a small group of students guided by a tutor confront a problem using a structured “7 jumps approach”: 1) clarify unfamiliar terms; 2) define problems; 3) brainstorm for possible explanations; 4) arrange tentative solutions and assign priorities; 5) formulate focused, achievable, comprehensive and appropriate learning objectives; 6) undertake private study; and 7) share and evaluate results [10–12]. In order to ensure a systematic approach to curriculum development, the well-known Dick and Carey model for instructional design [25] was adopted. The output from one phase of the model provided the input for the next phase, and the process included: analysis of learning needs; design / development of learning objectives, as well as instruments and materials for assessment; design of participants’ assessment with increased emphasis on formative evaluation; and evaluation of the whole instructional strategy and delivery formats while they were being designed [25].

### Needs assessment and PBL curriculum development

In February 2017, 11 health professionals (three pediatricians, five midwives, two nurses and a graduate in education) were selected by the FVG Regional Health Authority as members of the working group in charge of curriculum design. The meetings for curriculum construction took place from May to September 2017 and prioritized small group discussions with an action-oriented, participatory and supportive approach, allowing a wide involvement and commitment of the 11 professionals of the working group. The process was facilitated by two breastfeeding senior experts (AC, SQR), with the technical support of an expert in health professionals’ education (EPV). In parallel, an anonymous online survey was sent out to all the estimated 400 health professionals directly involved in the management of pregnant and / or breastfeeding women in the nine FVG maternity hospitals and the three LHAs, with no exclusion criteria. After explaining the objective of the survey, two pilot-tested, structured questions investigated frequent clinical difficulties faced during daily work and perceived breastfeeding CME needs, in addition to profession and place of work. The results of this online survey were used as input for the assessment of needs, the first phase of the Dick and Carey model [25].

### Training of PBL tutors

Between May and June 2017, the expert in health professional’s education (EPV) carried out three editions of a 3 − day introductory course on PBL principles for 32 health professionals, including the 11 professionals involved in curriculum development. These, a group of pediatricians, gynecologists, midwives and pediatric nurses from hospitals and LHAs, were selected by the FVG Regional Health Authority based on previous in-service training experience, using the WHO / UNICEF courses, and on willingness to learn and practice the PBL approach. The objectives were to introduce the PBL approach to future tutors and to simulate practical sessions and tutorial groups using the “7 jumps”, under supervision. During these introductory courses, participants performed an analysis of the FVG context and learners’ characteristics for the upcoming PBL curriculum, using a focus group approach. A summary of the focus groups results was provided as data input to the working group on curriculum design, as recommended by the Dick and Carey model [25]. In the second half of 2017, the same working group was also in charge of the development of training materials and assessment tools.

### PBL courses

PBL courses started in November–December 2017 and continued through 2019; only two courses were conducted in 2020 due to the Covid-19 pandemic. All participants were asked to fill in a course evaluation questionnaire at the end of each course. The questionnaire explored with a 5 − point Likert scale the perceived effectiveness of the course, its relevance to practice, and the overall educational quality.

### KAP survey

Health professionals who attended the PBL courses in 2019 were asked to complete an online survey to assess knowledge, attitudes and practices (KAP) of participants just before the course (T0), soon after it (T1), and 4–6 months later (T2). The KAP questionnaire included 20 multiple choice questions on knowledge, ten on attitudes and five on practices. A content validity was assured by a multi-professional group including two breastfeeding senior experts, two pediatricians, two CME experts, one gynecologist with expertise in health professionals’ education, one neonatologist, and one epidemiologist. Knowledge questions, with only one right answer out of five, were assigned a score of 1 in case of a correct answer and a score of 0 otherwise. Attitude and practice questions had answers in a 5 − point Likert Scale, ranging from ‘full agreement’ to ‘full disagreement’ for attitudes, and from ‘always’ to ‘never’ for practices.

### Statistical analysis

In the analysis of the KAP questionnaire, a score of 1 was given to the two answers closer to the correct one and a score of 0 to the remaining ones, except for one practice question for which there was only one right answer. In line with other similar surveys [26], missing answers were scored as incorrect. Adding up the results obtained for each question, a separate score for knowledge, attitudes and practices and an overall KAP score were calculated. Differences between T0 and T1, T0 and T2, and T1 and T2 were evaluated for each single dichotomized question with the McNemar test and for the KAP scores with the Wilcoxon signed ranks test. Independent comparisons between times were made and consequently no correction for multiplicity was carried out.

### Ethical aspects

This project did not imply any clinical experiment or intervention in human subjects, and a formal ethical clearance was considered unnecessary. For the online surveys, the participation was voluntary. Objectives and methods were explained in a written form to each health professional invited to participate, and an informed consent was sought before participation. Data collected were anonymized and no detail is reported that could reveal the identity of participants.

## Results

### Needs assessment

**Fig. 1:**
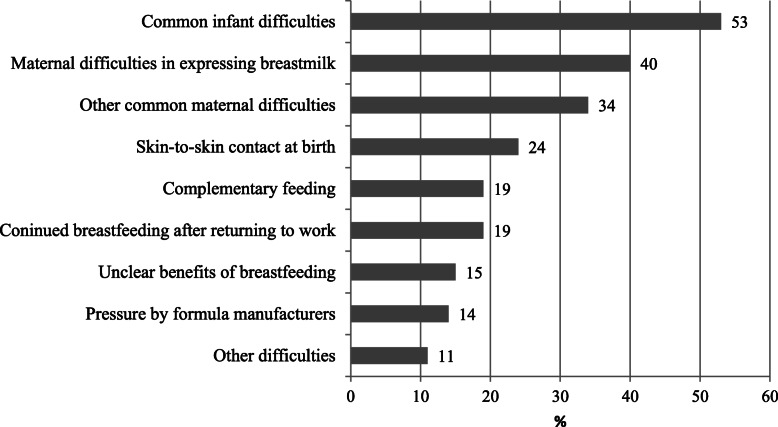
Practical difficulties identified by health professionals in the online survey. Percentage; total over 100% due to multiple answers

**Fig. 2:**
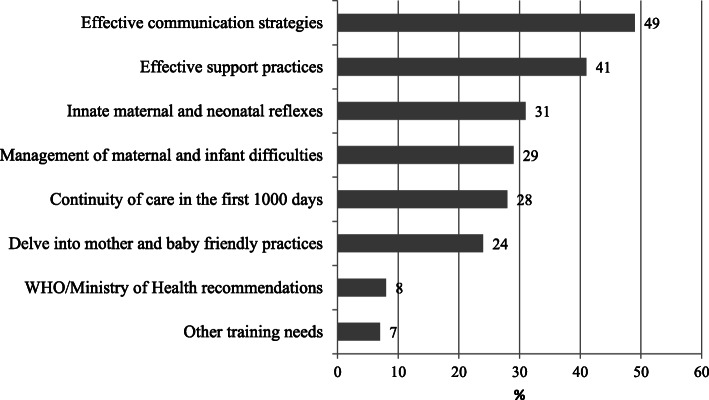
Learning needs identified by health professionals in the online survey. Percentage; total over 100% due to multiple answers

A total of 343 health professionals, out of 400, filled in the online survey between May and June 2017: 142 midwives (41%), 110 nurses (32%), 56 pediatricians (16%), 15 gynecologists (4%), and 20 other professionals (6%); 83% of the health professionals involved were working in maternity hospitals. Figure 1 shows the key practical difficulties identified by respondents. Among common infant difficulties, perceived breastmilk insufficiency, low weight gain and excessive crying were the most cited. Difficulties in expressing or pumping breastmilk were prevalent among maternal challenges, followed by sore or cracked nipples. The management of skin-to-skin contact immediately after birth for early initiation of breastfeeding was also seen as a major difficulty. Figure 2 shows the answers to the question on learning needs. The need for effective communication with mothers and families was the most cited, followed by the use of effective support practices, by a better understanding of innate maternal and neonatal behaviours that favour initiation of breastfeeding, and by the need to improve management of common maternal and infant difficulties. Continuity of care during the first 1000 days was also seen as a prominent need. These results, and the results of the above-mentioned focus group discussions with future tutors, were used to identify the learning objectives for the development of the curriculum.

### PBL curriculum

The process of curriculum design resulted in an inter-professional course on the protection, promotion and support of breastfeeding in FVG. This is a 29 − hour course to be delivered in four days over four consecutive weeks. Tutorial group topics based on real problems and practical activities, including simulations and role plays, were given priority: 36 and 35% respectively of the total time. Other activities included supervised clinical practice in a maternity hospital or community health service (16% of total time), three introductory lectures (8%), and two hours of miscellaneous activities such as introduction of participants and group feedback plenaries (6%). The curriculum is vertically and horizontally integrated, i.e., across time and across disciplines, with a progression from simple to complex, and from physiological to pathological issues. Themes such as ethics (including compliance with the International Code) [27], communication and woman-centred clinical management run throughout the four days and all activities as pillars of inter-professional and integrated training. Table 1 shows the main innovations introduced in the new breastfeeding curriculum mainly based on the PBL approach.

**Table 1:** Innovations in the PBL curriculum for the course on protection, promotion and support of breastfeeding

Learning objectives	• Defined after a local needs assessment
	• Broken down by knowledge, skills and attitudes
	• Priority to the respect for physiology
	• Centered on women, empowerment and continuity of care
	• Supported by pillars: ethics, communication, clinical practice
Participants	• Maximum 24 participants per course considering availability of physical structure and human resources for three tutorial groups working in parallel
	• Maximum 8 participants per tutorial group and 4 per practical session to stimulate interaction among participants, enhancing group discussion and learning
	• Different professional background and service affiliation in small groups discussions to favour sharing of knowledge and experiences and enhance learning outcomes
Learning activities and materials	• Priority to tutorial groups and practical sessions
	• Manuals for tutors and participants provided at the beginning of the course
	• Hard copies of lectures and set of slides provided beforehand
Assessment procedures	• Criteria clearly discussed with participants at the beginning of each course
	• Dedicated checklists available in the participant’s manual
	• Higher weight to tutorial groups and practical session assessments in the final score
	• Daily assessment of contributions to construction of group knowledge, performance during group work and support to other participants
	• Daily formative evaluation: frequent constructive feedback
	• Written exam with combined multiple choice and short answer questions
	• Follow up programme for participants who fail

### Learning materials

To facilitate the tasks and performance of tutors and participants, to make the whole process more transparent, and to show the links among all the activities included in the curriculum, three manuals were developed de novo:

1. A 41 − page manual for tutors including glossary, educational objectives, course schedule and detailed guides for each of the three tutorial groups, the seven practical activities and the supervised clinical practice (including tips to enhance group discussions). In addition, as supportive information, the manual includes instructions for the assessment and formative evaluation of participants, a checklist on communication skills, a list of suggested positive attitudes for tutors, a list of references for PBL and breastfeeding, a suggested constructive feedback model, and the three ready-made PowerPoint presentations. As examples, cases prepared ad-hoc for tutorial groups are presented as Additional file 1.
2. A 44 − page manual for practical activities and the supervised clinical practice in which tutors can find support materials such as clinical cases for the tutorial groups with specific learning objectives, instructions for checklist use, tables, pictures, drawings, extracts from the literature, and suggested solutions and tips for conducting role-plays, as well as suggestions for constructive feedback.
3. A 50 − page manual for participants with contents taken from the previous two manuals, condensed or expanded as appropriate.

Table 2 lists the topics integrated into the course.

**Table 2:** List of topics integrated into the PBL course

• Physiological changes during pregnancy, birth and breastfeeding	
• Physiology of breastfeeding, including primitive innate maternal and neonatal reflexes	
• Benefits of skin-to-skin contact and zero separation	
• Main breastfeeding difficulties and support strategies	
• Introduction of complementary foods	
• The 10 and 7 Steps of the Baby Friendly Hospital and Community Initiatives	
• The WHO / UNICEF recommendations on breastfeeding	
• Breastmilk substitutes and the International Code	
• The influence of marketing on infant and young child feeding	
• Continuity of care between hospital and community services	
• Effective communication with and empowerment of families	
• The rights of working mothers and their protection	

### Courses

The first pilot breastfeeding course was run in November–December 2017. The first invited participants were the professionals who had attended the courses on PBL in May–June 2017, i.e., the future regional tutors. The main purpose was to field test the curriculum, timetable, manuals and learning materials, while simultaneously providing a PBL training opportunity to future tutors and some evidence-based updates on breastfeeding. The field test was considered positive as far as curriculum, schedule, manuals and materials were concerned; only minor modifications were deemed necessary. Out of 24 participants, 21 accepted to become tutors, in addition to the 11 initially selected to develop the curriculum. These 32 regional tutors started to run courses in different hospitals and LHAs from mid-2018. By the end of 2018, the tutors had conducted 20 courses with 312 participants; 15 more courses for 223 participants were held in 2019, and two with 27 participants in 2020, for an overall total of 562 trainees, many more than the 400 initially planned.

### PBL course evaluation

**Fig. 3:**
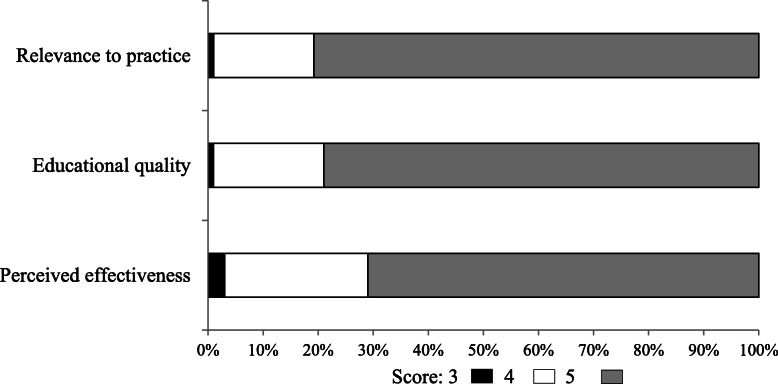
Proportional distribution of the scores (scale: 1 to 5) assigned to relevance to practice, educational quality, and perceived effectiveness by 519 course participants in 2018–2020 courses. Scale 1 to 5 (1 = lower score; 5 = higher score); percentage of participants within bars

The course evaluation questionnaire was filled in by 519 (92.3%) participants in the period 2018–2020. Figure 3 shows the distribution of the scores assigned to relevance to practice, educational quality, and perceived effectiveness. Only a limited number of participants assigned a low score (3 or less). Most participants gave their course a score of 4 or 5 (98, 99 and 97%, respectively).

### KAP survey

The online KAP questionnaire was proposed to participants of nine courses in 2019. Complete answers to the T0, T1 and T2 questionnaires were received from 105 / 137 participants (76.6%): 20 doctors (eight pediatricians and 12 gynecologists), 44 midwives, 27 nurses (three of which were pediatric nurses), and 14 other professionals. Table 3 shows the overall scores. There were statistically significant differences between T0 and T1 and between T0 and T2, but not between T1 and T2, indicating a general improvement after the course and good retention of knowledge, attitude and practice after 4–6 months. The detailed analysis of the scores obtained for each question showed a statistically significant improvement between T0 and T1 and between T0 and T2, with retention of a good score between T1 and T2, for 11 out of 20 knowledge questions, seven out of ten attitude questions, and two out of five practice questions. For the knowledge and attitude questions, those showing no statistically significant improvement were the ones for which more than 90% of participants had already ticked the right answer at T0. Among the five practice questions, the ones without statistically significant improvement referred to the duration of skin-to-skin contact soon after birth and the need to show all mothers how to hand pump breastmilk.

**Table 3:** Overall score for the KAP survey at T0, T1 and T2 (*n* = 105)

Score	Time			Wilcoxon signed ranks test		
	T0	T1	T2	p T1-T0	p T2-T0	p T2-T1
Knowledge						
25°	14.0	18.0	18.0	< 0.0001	< 0.0001	0.91
Median	17.0	19.0	19.0			
75°	18.0	19.0	19.0			
Attitude						
25°	7.0	9.0	9.0	< 0.0001	< 0.0001	0.90
Median	8.0	9.0	9.0			
75°	9.0	10.0	10.0			
Practice						
25°	1.0	1.50	2.0	0.002	0.001	0.50
Median	3.0	3.50	4.0			
75°	4.0	4.0	5.0			
Total						
25°	24.0	29.0	29.0	< 0.0001	< 0.0001	0.51
Median	27.0	31.0	31.0			
75°	30.0	32.0	32.0			

### Exclusive breastfeeding rates

The FVG reporting system on the prevalence of breastfeeding at discharge from maternity hospitals and at four to five months of age showed an increase in the rates of exclusive breastfeeding between 2017 and 2019, allowing for EBF at 4–5 months to recover the loss suffered from 2010 to 2016 (Table 4).

**Table 4:** Rates of exclusive breastfeeding in FVG between 2017 and 2019

Year	Exclusive breastfeeding			
	At discharge from maternity ward		At four to five months of age	
	n / N	%	n / N	%
2017	5910 / 7896	75	1640 / 5323	31
2018	6301 / 7795	81	1891 / 5688	33
2019	6158 / 7625	81	2077 / 5581	37

## Discussion

To our knowledge, this is the first time the PBL approach has been used for short in-service training courses on breastfeeding. In FVG, this approach has been eagerly accepted by the selected tutors and greatly appreciated by participants (Fig. 3), despite some predictable shortcomings. It was in fact difficult to schedule a large number of courses that would keep health professionals away from their clinical duties for four days and the formal involvement of the FVG Regional Health Authority was crucial to facilitate the participation of tutors and health professionals. Sometimes it was even difficult to make the right locations available due to the number of rooms needed for small group work. In addition, participants needed to actively study the topics included in the course not only during it, but also as homework, a burden that many health professionals would find too heavy. Also, the course implied a burden of extra work, not easily quantifiable in terms of working hours, for tutors and for the staff in charge of CME accreditation, a time consuming but unavoidable administrative task in Italy. Yet, the results achieved so far, in terms of training coverage, satisfaction and acquisition of knowledge and skills, are encouraging.

An important positive outcome of this project was the establishment of a well-trained and experienced multi-professional team of tutors that is now available to FVG health authorities for scaling up of the PBL approach to other CME topics. The tutors trained for this project would be able to develop and run other courses, for example on complementary feeding, on nutrition for pregnant women, children and adolescents, on pre- and peri-natal health, or on other maternal and child health issues. A strength of this project was the adoption of inter-professional education, in which two or more professionals learn about, from and with each other to enable effective collaboration and improve health outcomes [19–21]. During our project, trainees progressed from independent work based on professional knowledge to collaboration with other professionals and to inter-professional thinking, as also described in literature [28].

The PBL approach applied to in-service training on breastfeeding showed a sustained improvement in knowledge, attitude and practice among participants. The longterm objective of increasing EBF rates and duration is ongoing, based on the routine data gathered by the FVG monitoring system. This shows an increase in the rates of EBF after 2018. Whether this improvement is due to upgrading on health professionals’ training related to the implementation of the PBL project or to some other factors, and whether it is greater, if really associated with PBL courses, than the one reported after courses developed for the BFHI, is a matter for further research. The BFHI courses are effective in increasing breastfeeding rates [29], but in Europe only a handful of countries have a significant number of designated BFHI hospitals and breastfeeding rates fall short of what is recommended [30].

A weakness of this project is related to the rapid turnover of staff in maternity hospitals and LHAs; health care services and departments will quickly be staffed by professionals not updated on breastfeeding, or other topics. But this weakness is common to all CME programmes. In the case of breastfeeding, it could be partly overcome by the assignment of a substantial number of teaching hours during pre-service training. Unfortunately, and with rare exceptions [31], schools for doctors, midwives and nurses in Italy, and elsewhere, do not generally assign the right priority to breastfeeding in their curricula, let alone adopt PBL. The Italian BFHI recognizes pre-service training programmes, in addition to hospitals and LHA. So far, only four programmes were accredited, three for midwives and one for nurses; none for doctors [32].

## Conclusions

Our results, if confirmed and consolidated, may have important implications for policy- and decision-makers. Conventional CME activities are known to be medium to poorly effective as far as knowledge, performance and patients’ outcomes are concerned; effectiveness may improve with interactive multiple methods, designed for small groups [33]. PBL responds to this indication. To our knowledge, there is no previous experience on breastfeeding, or on infant and young child feeding training courses, to compare with. Future studies should better explore possible enablers and barriers for the implementation of a similar project and should include a comprehensive evaluation of the effects on breastfeeding rates.

## Supplementary Information

**Additional file 1.** Cases prepared ad-hoc for tutorial groups

## Data Availability

The datasets used and analysed during the current project is available from the corresponding author on reasonable request.

## Abbreviations

BFHI: Baby Friendly Hospital Initiative
CME: Continuing Medical Education
EBF: exclusive breastfeeding
FVG: Friuli Venezia Giulia
KAP: Knowledge, Attitudes and Practices
LHA: Local Health Authority
PBL: Problem-Based Learning
UNICEF: United Nations Children’s Fund
WHO: World Health Organization
