# Supplementary material for: Problem-based learning for in-service training on breastfeeding in Friuli Venezia Giulia, Italy
Source: Int Breastfeed J. 2021 Nov 27;16:89. doi: 10.1186/s13006-021-00439-4 (PMC8626965; doi:10.1186/s13006-021-00439-4)
Supplement: Supplementary file 1 — Additional file 1. Cases prepared ad-hoc for tutorial groups [file 13006_2021_439_MOESM1_ESM.docx]

**Additional file 1 -** **Cases prepared ad-hoc for tutorial groups**

**Case 1. A second opportunity**

Christine is the second daughter of a female doctor who does not refer health problems during pregnancy, beyond the expected physiological changes. Christine is 48 hours old and fights with her mother to try to latch onto the breast. The mother reports that breastfeeding “was a battle” even with her first child, which led her to interrupt breastfeeding in the second month. In the delivery room, during skin-to-skin contact, the staff recommended to breastfeed in a semi-reclined position. In the ward, the mother preferred to leave the baby in the crib and never held her in her arms, except to breastfeed, for fear of “spoiling the baby”. Given the protracted difficulties of attachment, the semi-reclined position was re-proposed, which the mother did not accept because she considered it “a torture for the baby who risks suffocation.” Actually, Christine tried to re-attach to the breast through obvious attempts that her mother does not seem to recognize as such. At this point, the mother, hanging up the triangle of her bra, says very nervously and crying: “You don’t seem to be enough hungry. Try to sleep!”. The nurse, instead, caught in Christine’s behavior attempts to latch on to the breast and began to tell the mother about the innate abilities of newborns, expressed through primitive neonatal reflexes.

Learning objectives:

- To recognize the physical, cognitive and emotional changes of the woman during pregnancy, at birth and after birth.
- To explain the physiology of breastfeeding (hormones, composition and importance of colostrum and breast milk).
- To explain the importance of zero separation and skin-to-skin contact, in safe conditions, for mother and baby, regardless of the mode of delivery and the mother’s intentions on how to feed the baby.
- To interpret the maternal and neonatal innate reflexes after birth and their importance in initiating and maintaining breastfeeding, in the relationship of attachment and care, and in the mutual regulation of behavior.

**Case 2. Unexpected arrival**

My pregnancy went very well, but Francis’s birth found me unprepared. He wanted to see the world at 37 weeks, broke the waters, in 3 hours he was born. He was so small, 2,300 gr. I didn’t hear him cry. They took him away immediately because he was not breathing well. For a few days he was in a thermal cradle and I was told that he had respiratory distress. The first time I saw him he was three hours old, the nurse was piercing him in the heel and he had a tube that went from his mouth to his stomach. I had so much wanted to keep him with me right away and breastfeed him, but on the days he was in the crib I could only stroke him for a few minutes. The first time I held him he was already three days old. He was still losing weight and I had little milk, a small drop of a yellowish milk, perhaps for this reason he showed little interest in my breasts. They recommended a breast pump, luckily I had bought one advised by my mother-in-law, and they called me to give him milk with a bottle. Then I started to produce milk, but when Francis latched on he made me feel an sharp pain. The pain continued and for a few days my breasts became tight, hot and red. I tried everything, including the “miraculous” cups I had seen in TV. But there was no way. Francis is now three weeks old and we are at home, he cries a lot and I am very tired. He grows little and I think I don’t have enough milk. I’m about to offer him a supplement of formula.

Learning objectives:

- To identify the conditions in which mother and baby need help with breastfeeding and/or need clinical evaluation and/or treatment:
  - conditions of the mother: for example, pain in the breast and nipple, mastitis;
  - conditions of the newborn: for example, prematurity, respiratory distress, hypoglycemia, difficulty in latching and sucking, poor growth.
- To plan adequate strategies to protect breastfeeding in case of separation between mother and baby or during the treatment of clinical situations that may compromise breastfeeding:
  - early start of breastfeeding, risks of delayed breastfeeding;
  - how to establish the supply of breastmilk in case of separation and with what methods (frequency and advantages of manual expression vs breast pump).
- To describe the medical indications for supplementation with formula.
- To understand the impact of marketing on the practices of health professionals and families in terms of health and nutrition of mothers and children.

**Case 3. Background noise**

Ann and Mark, 4 months ago, were delighted by the birth of their first baby. Ann is breastfeeding her exclusively and with satisfaction, 7/8 times a day. She will soon resume work, and she will have to leave her daughter at the nursery. Her employer has informed her that she will have to start over full time (8 hours), but her husband claims that this is an improper request. Ann is very concerned about how she will be able to continue feeding her baby. She talked about it with her friends who suggested she switch to formula, along with a well-known baby food advertised as specific for the first phase of complementary feeding. Ann, however, fears that her baby be not ready yet. The aunt, on the other hand, advises her to rent a pump, thus allowing those who stay with the baby to feed her with a bottle. Ann, increasingly confused, turns to the counseling center of her health district.

Learning objectives:

- To summarize the rights of parents during pregnancy, in the first months of the child’s life and when the mother returns to work.
- To analyze the effects of an improper use of pacifiers, nipple shields and breast pumps.
- To list the risks of formula feeding, improper use of teats, baby bottles and industrial foods.
- To recognize the importance of introducing complementary foods when the infant is ready.
- To understand the impact of marketing on the practices of professionals and families in terms of health and nutrition of mothers and children.
